# Associations between GJB2, Mitochondrial 12S rRNA, SLC26A4 Mutations, and Hearing Loss among Three Ethnicities

**DOI:** 10.1155/2014/746838

**Published:** 2014-04-02

**Authors:** Wan Du, Qiuju Wang, Yiming Zhu, Yanli Wang, Yufen Guo

**Affiliations:** ^1^Department of Otolaryngology-Head and Neck Surgery, The Second Hospital of Lanzhou University, Lanzhou 730030, China; ^2^Department of Otolaryngology-Head and Neck Surgery, Chinese People's Liberation Army Institute of Otolaryngology, Chinese People's Liberation Army General Hospital, Beijing 100039, China; ^3^Health Department of Gansu Province, Lanzhou 730030, China

## Abstract

The epidemiological researches show that the mutations of GJB2, mitochondrial 12S rRNA, and SLC26A4 genes have played an important role in the hearing loss. This study aims to investigate the mutation spectrum of GJB2, mitochondrial 12S rRNA, and SLC26A4 genes of Han Chinese, Hui people, and Uyghur ethnicities in sensorineural hearing loss (SNHL) patients in northwest of China. Mutational analyses in the three genes were brought by direct sequencing and each fragment was analyzed using an ABI 3730 DNA Sequencer. The mutation frequencies for the three HL causative genes were 34.05% in Han Chinese participants, 27.47% in Hui people, and 14.44% in Uyghur participants, respectively. The prevalence of GJB2 mutations was 13.7%, 11.4%, and 11.4% in Han Chinese, Hui people, and Uyghur participants (*χ* ^2^ = 10.2, *P* < 0.05), respectively. The prevalence of mtDNA 12S rRNA A1555G homozygous mutations was 6.05%, 3.27%, and 1.44% in Han Chinese, Hui people, and Uyghur participants (*χ* ^2^ = 13.9, *P* < 0.05), respectively. The prevalence of SLC26A4 mutations was 14.3%, 12.8%, and 1.6% in Han Chinese, Hui people, and Uyghur participants, respectively. In summary, we find that Uyghur and Hui SNHL individuals vary significantly from Han Chinese patients in three causative HL genes' mutational spectrum, especially for Uyghur.

## 1. Introduction

Hearing loss (HL) is not only a common cause of human speech disorder, but also an essential factor affecting the population's quality of life. Current studies have shown that newborns of one in 1000 are diagnosed as congenital HL [1–3]. And this disease is mainly caused by genetic factors. Among them, nonsyndromic hearing loss (NSHL; hearing loss is the only symptom) is accounted for over 70% of elements. Its divergent patterns include autosomal dominant (DFNA), autosomal recessive (DFNB), X-linked recessive, and mitochondrial heritance. Up to now, more than 130 genetic loci and 80 genes have been described for NSHL (http://hereditaryhearingloss.org/, updated in August 2013). The epidemiological researches show that the mutations of GJB2, mitochondrial 12S rRNA, and SLC26A4 genes have played an important role in the occurrence of HL [4–7].

Mutations in GJB2 are considered to be in charge of about 50% of genetic sensorineural HL, whereas the patterns of GJB2 mutations are recognized to be related to the ethnic background. The most common mutations of GJB2 among Caucasian, Jewish, and Asian populations are c.35delG, c.167delT, and c.235delC, respectively [8–15]. Mitochondrial 12S rRNA is delivered by the mother. Mutations in this gene are associated with aminoglycoside-induced HL. SLC26A4 gene accounts for about 1%*∼*12% reason of sensorineural HL in children. So far, over 200 mutations have been identified in this gene. In different ethnicities or countries, specific variants of the gene were found in various studies.

Northwest of China (Shaanxi, Gansu, Qinghai, Ningxia, and Xinjiang provinces) is the corridor of connecting Asia and Europe which is known as “Silk Road.” In the past, this region witnessed the migration of population resulting in the mixture of people in the east and west. It brings the abundant genetic background and makes the region become a multiethnic gathering place, mainly populated by Hui people and Uyghur ethnicities. This study aims to investigate the mutation spectrum of GJB2, mitochondrial 12S rRNA, and SLC26A4 genes of Han Chinese, Hui people, and Uyghur ethnicities in sensorineural hearing loss (SNHL) patients. And it may also provide the scientific basis for diagnosis, therapy, and genetic counseling of HL in minority ethnic people.

## 2. Materials and Methods

### 2.1. Study Population and Data Collection

A total of 2349 unrelated sensorineural hearing loss individuals (case group) from northwest of China were recruited in this study at the Department of Otorhinolaryngology Head and Neck Surgery in the Second Affiliated Hospital of Lanzhou University and the Chinese People's Liberation Army General Hospital. As a control group, 655 normal hearing individuals were enrolled from the same region. The above subjects volunteered to give informed consent by themselves or their legal guardians prior to their participation in this research. The research protocol was approved by the Ethics Committee of Chinese People's Liberation Army General Hospital.

Comprehensive clinical history and physical examination were performed on each participant to identify any syndromic findings. Audiological examinations were performed including pure-tone audiometry (PTA), auditory brainstem response (ABR), distortion product otoacoustic emissions (DPOAE), and immittance testing. According to the World Health Organization (WHO) standard in 1997, the severity of hearing loss was classified into five ranks: normal <26 decibel (dB); mild = 26–40 dB; moderate = 41–70 dB; severe = 71–90 dB; and profound >90 dB (using PTA from the average of the audiometric thresholds at 500, 1000, 2000, and 4000 Hz to calculate). Subjects with binaural moderate to profound HL were included. At the same time, investigators excluded middle ear disorders and syndromic HL. For members of HL pedigree, one person in this pedigree was chosen as a proband to analyze. A high-resolution computed tomography (HRCT) scan of petrous temporal bone was obtained for evaluation of SLC26A4 mutations leading to large vestibular aqueduct syndrome (LVAS). Finally, 10 mL peripheral blood samples were taken from the participants.

### 2.2. DNA Separation and Mutational Analysis

Firstly, genomic DNA was isolated from peripheral leukocytes. Then, subjects' DNA fragments were amplified by PCR. The amplified segments of GJB2, mtDNA 12SrRNA, and SLC26A4 genes were purified with a Montage PCR 96 Millipore plate. After that, each fragment was analyzed using direct sequencing in an ABI 3730 DNA Sequencer (Applied Biosystems, Foster City, CA, USA). Finally, the sequence data were analyzed with the reference sequences in NCBI (NG_008358 for GJB2, NC_012920 for mtDNA 12S rRNA A1555G, and NG_008489 for SLC26A4) by the DNAStar 7.0 software. Mutations or polymorphisms were compared with the reference sequences.

### 2.3. Statistical Analysis

The statistical analysis was performed by SPSS 17.0 software. Rates of mutation were compared using multiple-sample chi-square test among Han Chinese, Hui people, and Uyghur ethnic groups. When there was no difference, two-sample chi-square tests were assumed as significant at *P* values ≤0.05.

## 3. Results

In this research, the case group (*n* = 2349) consisted of 1835 Han Chinese participants (with the age range of 1–39, 13.6 ± 4.0 years, female/male ratio of 798/1037), 306 Hui people (with the age range of 2–28, 14.4 ± 4.3 years, female/male ratio of 136/170), and 208 Uyghur participants (with the age range of 4–22, 13.8 ± 3.7 years, female/male ratio of 110/98). Audiological assessments indicated that all of 2349 patients had moderate to profound SNHL (99% of cases were profound ones). Among them, 178 cases had a family history of HL (137 Han Chinese, 32 Hui people, and 9 Uyghur individuals). The rest of the 2170 cases were considered to be sporadic. As control subjects, 655 normal hearing samples who came from the same region as case group were recruited including Han Chinese (*n* = 300), Hui people (*n* = 228), and Uyghur ethnicities (*n* = 127). The frequencies of mutations for three deafness-associated genes in the three ethnicities were revealed in Table 1.

### 3.1. Mutation Analyses of GJB2, Mitochondrial 12S rRNA A1555G, and SLC26A4

The prevalence of GJB2 mutations was 13.7%, 11.4%, and 11.4% in Han Chinese, Hui people, and Uyghur participants (*χ* ^2^ = 10.2, *P* < 0.05), respectively. The allele distributions and frequencies including four common mutations in three ethnicities were presented in Table 2, namely, c.235delC, c.299_300delAT, c.176_191del16, and c.35delG. Among them, c.235delC showed the highest frequency and the mutation rates were significantly different in three ethnicities (*χ* ^2^ = 17.3, *P* < 0.05). Meanwhile, the prevalence of c.35delG mutation which is the hotspot mutation in Caucasian population was higher in Uyghur than in Hui people and Han Chinese (*χ* ^2^ = 30.6, *P* < 0.05). The prevalence of mtDNA 12S rRNA A1555G homozygous mutations was 6.05%, 3.27%, and 1.44% in Han Chinese, Hui people, and Uyghur participants (*χ* ^2^ = 13.9, *P* < 0.05), respectively. And the allele frequency of mtDNA 12S rRNA A1555G was shown in Table 3. The prevalence of SLC26A4 mutations was 14.3%, 12.8%, and 1.6% in Han Chinese, Hui people, and Uyghur participants, respectively. The allele frequencies including ten mutations in the three ethnicities of SLC26A4 gene were pointed out in Table 4. Observably, c.919-2A>G (IVS7-2A>G) and c.2168A>G (H723R) were the main mutant patterns in the above ethnicities. The allele frequency of c.919-2A>G was notably higher in Han Chinese than in Uyghur (*χ* ^2^ = 20.9, *P* < 0.05), whereas there was no significant difference in Han Chinese and Hui people (*χ* ^2^ = 2.8, *P* > 0.05). Simultaneously, the allele frequency of c.2168A>G was also higher in Han Chinese than in Uyghur (*χ* ^2^ = 4.6, *P* < 0.05) and there was no significant difference between Han Chinese and Hui people (*χ* ^2^ = 0.01, *P* < 0.05). The above mutations were not found in a screen of 655 normal hearing individuals.

## 4. Discussion

As shown in this research, the mutation frequencies for the three HL causative genes were 34.05% in Han Chinese participants, 27.47% in Hui people, and 14.44% in Uyghur participants, respectively. There were significant differences in mutation frequencies between Han Chinese and minority population. The allele frequencies were significantly lower in control group than in case group for either ethnicity. Ethnicity was a significant factor contributing to mutational form in the occurrence of HL. It indicated that the etiology of HL in the minority population needed further analysis of other HL-related genes, especially for the Uyghur patients.

### 4.1. GJB2

GJB2 gene encodes protein connexin 26 which may have an effect on K+ circulation in the cochlea. Mutations in GJB2 are the most common reasons for a large proportion of SNHL in China and other countries. Previous studies found that the prevalence of GJB2 variants varied among different ethnic groups. Results from this research showed that the mutation frequencies of GJB2 in Han Chinese, Hui people, and Uyghur patients were 13.7%, 11.4%, and 11.4%, respectively. Dai et al. showed that the prevalence of GJB2 mutation was about 4%*∼*30% in Chinese nonsyndromic HL individuals [16]. There was no significant difference between our research and the study of Dai et al. In addition, the mutant loci and allele frequencies varied significantly among the three ethnicities. Among the three common mutations of GJB2 detected in Han Chinese participants, c.235delC showed the highest allele frequency, followed by c.299_300delAT. This is consistent with the fact that the c.235delC mutation is found to be the most common mutation in Eastern Asians [17, 18]. For Uyghur, c.235delC also showed the highest allele frequency, followed by c.35delG. The c.35delG is the chief mutation in Caucasians [19]. Both c.235delC and c.35delG were simultaneously detected as the predominant mutations in Uyghur participants that probably resulted from the ethnic origin of Uyghur people. Located near Central Asia, Uyghur people have merged 30% of Caucasian kinship via the migration and cross-ethnic marriage, making themselves the Caucasian and Mongolian features simultaneously [20]. For Hui people, the results showed that the prevalence of c.235delC and c.35delG was between Han Chinese and Uyghur.

### 4.2. mtDNA 12S rRNA A1555G

The mtDNA 12S rRNA, matrilineal mitochondrial gene, is related to aminoglycoside-induced HL [21]. Among the multiple variants, m.1555A>G is the more commonly detected one which shows an alterable prevalence resulting from the ethnic variation [22]. In the Asian SNHL population, previous reports showed that the incidence of the m.1555A>G mutation seemed to be higher in Caucasians [23–26]. Results from this study indicated that the allelic frequency was 5.5% for Han which was significantly higher than Hui and Uyghur. The difference of the variant frequency in m.1555A>G mutation among three ethnicities lies in geographical and environmental differences. Given that the racial factors influence gene mutation, Uyghur population has their roots in Caucasians and the genetic makeup is similar to the Caucasians. The Hui ethnicity that migrated from Persian and Arab worlds is one of the major minorities in China. The western Eurasian specific haplogroup frequency of mtDNAs in the Hui was 6.7%; however, no western Eurasian type was shown in Han samples from the same place [27].

### 4.3. SLC26A4

As the second most common causative HL gene in Chinese population, SLC26A4 mutation is related to the enlarged vestibular aqueduct and accounts for 4%*∼*15% of hereditary HL individuals [28]. In our study, the prevalence of SLC26A4 mutations was 14.3%, 12.8%, and 1.6% in Han Chinese, Hui people, and Uyghur participants, respectively. For Han Chinese, eight mutation types were found: c.919-2A>G, c.2168A>G, c.2162C>T, c.919-18T>G, c.920C>T, c.987A>G, c.2183A>G, and c.1001+32A>G. Different from Han, c.919-2A>G, c.2168A>G, c.2162C>T, c.1001+5G>C, c.1001+32A>G, and c.2107C>G were detected in the Hui people, while for Uyghur, only c.919-2A>G and c.2168A>G were found. Among the eight mutations of SLC26A4 detected in Han Chinese participants, c.919-2A>G (11.0%) showed the highest allele frequency, followed by c.2168A>G (3.1%). The frequency of c.919-2A>G and c.2168A>G in Uyghur was lower than those of Han Chinese. The low-frequency mutation in Uyghur SNHL participants is inconsistent with the fact that the SLC26A4 is the second most common causative HL gene in Chinese population. This result may be inferred from the different origins of the Han and Uyghur ethnicities. Whether Uyghur shares some common mutations in SLC26A4 with Caucasians or not needs to be researched. The research of mitochondrial DNA haplotype shows that modern Uyghur is the result of racial mixing between the east and west. Dissimilar to Uyghur, Han Chinese belongs to Mongolia. In North Europe, the common mutant loci of SLC26A4 are p.L236P, p.T416P, and IVS8+1G>A, while c.2168A>G is the main mutant locus in Eastern Asian (Korean and Japanese) HL patients. IVS7-2A>G, however, is found to be the primary mutant locus of SLC26A4 in Chinese mainland and Taiwan. There was a significant difference between Hui and Uyghur in the frequency of c.919-2A>G, whereas no statistical significances were found between Hui and Han in the frequencies of c.919-2A>G and c.2168A>G. The molecular biology studies show that there is considerable migration evidence between the Hui and Han [27, 29]. Therefore, in view of hereditary distance, Hui is closer to Han.

## 5. Conclusion

In summary, we found that Uyghur and Hui SNHL individuals varied significantly from Han Chinese patients in three causative HL genes' mutational spectrum, especially for Uyghur. Our findings also verified that there was an interethnic difference in the Han, Hui, and Uyghur population. Detection of GJB2, MT-RNR1, and SLC26A4 genes may only illuminate the molecular defects of about 34%, 27%, and 14% of the patients with SNHL in Han, Hui, and Uyghur, respectively. Moreover, other HL-associated genes are required to be examined in the remaining patients of unidentified inheritance defects.

## Figures and Tables

**Table 1:** Prevalence of three genes in three ethnicities.

Ethnicity	GJB2		mtDNA1555G		SLC26A4		Total		
	*n* of mutations	Rate	*n* of mutations	Rate	*n* of mutations	Rate	*n *	*n* of mutations	rate
Han Chinese	251	13.7	111	6.05	269	14.66	1835	631	34.39
Hui people	35	11.4	10	3.27	37	12.09	306	82	26.8
Uyghur	24	11.4	3	1.44	3	1.44	208	30	14.42

**Table 2:** GJB2 distribution in three ethnicities.

Ethnicity	Alleles (*n*)	c.235delC				c.299_300delAT				c.176_191del16				c.35delG			
		Homo	Hetero	Total (%)		Homo	Hetero	Total (%)		Homo	Hetero	Total (%)		Homo	Hetero	Total (%)	
Han Chinese	3618	115	132	362	73.3	14	50	78	15.8	0	12	12	2.4	1	8	10	2.0
Hui people	616	16	14	46	65.7	2	5	9	12.9	0	6	6	8.6	2	4	8	11.4
Uyghur	414	5	10	20	42.6	1	2	4	8.5	0	1	1	2.1	2	8	12	25.5

**Table 3:** mtDNA distribution in three ethnicities.

Ethnicity	Alleles (*n*)	m.1555A>G		Others	
		*N* (%)		*N* (%)	
Han Chinese	3618	198	5.5	24	0.7
Hui people	616	20	3.2	0	0
Uyghur	414	6	1.4	0	0

**Table 4:** SLC26A4 distribution in three ethnicities.

Ethnicity	Alleles (*n*)	c.919-2A>G *N* (%)	c.2168A>G *N* (%)	c.2162C>T *N* (%)	c.919-18T>G *N* (%)	c.920C>T *N* (%)	c.987A>G *N* (%)	c.2183A>G *N* (%)	c.1001+32A>G *N* (%)	c.1001+5G>C *N* (%)	c.2107C>G *N* (%)
Han Chinese	3618	386 (10.7)	112 (3.1)	12 (0.3)	2 (0.05)	2 (0.05)	1 (0.03)	1 (0.03)	1 (0.03)	0	0
Hui people	616	48 (7.8)	24 (3.9)	2 (0.3)	0	0	0	0	2 (0.3)	2 (0.3)	1 (0.2)
Uyghur	414	4 (1.0)	3 (0.7)	0	0	0	0	0	0	0	0
